# Skin Commensal Staphylococci May Act as Reservoir for Fusidic Acid Resistance Genes

**DOI:** 10.1371/journal.pone.0143106

**Published:** 2015-11-18

**Authors:** Wei-Chun Hung, Hsiao-Jan Chen, Yu-Tzu Lin, Jui-Chang Tsai, Chiao-Wei Chen, Hsiao-Hung Lu, Sung-Pin Tseng, Yao-Yu Jheng, Kin Hong Leong, Lee-Jene Teng

**Affiliations:** 1 Department of Microbiology and Immunology, Kaohsiung Medical University, Kaohsiung, Taiwan; 2 Department of Clinical Laboratory Sciences and Medical Biotechnology, National Taiwan University College of Medicine, Taipei, Taiwan; 3 Center for Optoelectronic Medicine, National Taiwan University College of Medicine, Taipei, Taiwan; 4 Division of Neurosurgery, Department of Surgery, National Taiwan University Hospital, Taipei, Taiwan; 5 Taipei First Girls High School, Taipei, Taiwan; 6 Department of Medical Laboratory Science and Biotechnology, Kaohsiung Medical University, Kaohsiung, Taiwan; 7 Department of Laboratory Medicine, National Taiwan University Hospital, Taipei, Taiwan; Rockefeller University, UNITED STATES

## Abstract

We analyzed the occurrence and mechanisms of fusidic acid resistance present in staphylococci isolated from 59 healthy volunteers. The fingers of the volunteers were screened for the presence of staphylococci, and the collected isolates were tested for resistance to fusidic acid. A total of 34 fusidic acid resistant staphylococcal strains (all were coagulase-negative) were isolated from 22 individuals (22/59, 37.3%). Examination of the resistance genes revealed that acquired *fusB* or *fusC* was present in *Staphylococcus epidermidis*, *Staphylococcus capitis* subsp. *urealyticus*, *Staphylococcus hominis* subsp. *hominis*, *Staphylococcus warneri* and *Staphylococcus haemolyticus*. Resistance islands (RIs) carrying *fusB* were found in *S*. *epidermidis* and *S*. *capitis* subsp. *urealyticus*, while staphylococcal chromosome cassette (SCC)-related structures harboring *fusC* were found in *S*. *hominis* subsp. *hominis*. Genotypic analysis of *S*. *epidermidis* and *S*. *hominis* subsp. *hominis* indicated that the *fus* elements were disseminated in diverse genetic strain backgrounds. The *fusC* elements in *S*. *hominis* subsp. *hominis* strains were highly homologous to SCC*fusC* in the epidemic sequence type (ST) 239/SCC*mec*III methicillin-resistant *S*. *aureus* (MRSA) or the pseudo SCC*mec* in ST779 MRSA. The presence of acquired fusidic acid resistance genes and their genetic environment in commensal staphylococci suggested that the skin commensal staphylococci may act as reservoir for fusidic acid resistance genes.

## Introduction

Fusidic acid is a steroid antibiotic that is used to treat skin infections caused by staphylococci in some countries [1]. The major target of fusidic acid is elongation factor G (EF-G), which is involved in protein synthesis [2–4]. Two major mechanisms of fusidic acid resistance have been reported. One mechanism is alteration of the drug target site, which is due to mutations in *fusA* (encoding EF-G) or *fusE* (encoding ribosome protein L6) [2, 5, 6]. The other mechanism is protection of the drug target site, which is mediated by the FusB-family proteins [7–10]. The FusB proteins bind to EF-G on the ribosome, thereby allowing the dissociation of stalled ribosome·EF-G·GDP complexes that form in the presence of fusidic acid [3, 4, 6, 9, 10]. As a result, the ribosome clearance mediated by the FusB-family proteins rescues the stalled translation [6, 9, 10].

The FusB-family proteins are encoded by the *fusB*, *fusC*, *fusD* or *fusF* gene and usually cause low levels of fusidic acid resistance [7, 8, 11]. The *fusB* gene has been found in *Staphylococcus aureus* and other staphylococcal species, either carried on a plasmid [12, 13] or on phage-related resistance islands (RIs) integrated into the chromosome [14–16]. The *fusC* gene has been found in the staphylococcal chromosome cassette (SCC), such as SCC*fusC* [17], SCC_476_ [18], SCC*mec*_N1_ [19] and pseudo SCC*mec* [20]. The *fusD* and *fusF* genes are found exclusively in the chromosome of *Staphylococcus saprophyticus* and *Staphylococcus cohnii* subsp. *urealyticus*, respectively, which explains the intrinsic fusidic acid resistance of both organisms [7, 11].

Coagulase-negative staphylococci (CoNS), which constitute a major element of the commensal microflora of human skin, comprise a multitude of species including *Staphylococcus capitis*, *S*. *cohnii*, *Staphylococcus epidermidis*, *Staphylococcus haemolyticus*, *Staphylococcus hominis*, *S*. *saprophyticus* and *Staphylococcus warneri* [21–23]. CoNS have been identified as playing an important role as reservoirs of gene pools, which can facilitate pathogen infection. For example, *S*. *epidermidis* and *S*. *haemolyticus* may act as a source of the SCC*mec*, thereby allowing *S*. *aureus* to become methicillin-resistant *S*. *aureus* (MRSA), which is responsible for several difficult-to-treat infections [24, 25]. As another example, horizontal transfer of the arginine catabolic mobile element (ACME) from *S*. *epidermidis* to MRSA USA300 may provide multiple fitness advantages [26].

The rate of resistance to fusidic acid in staphylococci varies in different countries. For *S*. *aureus*, fusidic acid resistance rates ranged from 1.4% to 52.5% in European countries [27], 7% in Canada and Australia [28] and <0.35% in the United States [28, 29]. In Asian countries, the fusidic acid-resistant *S*. *aureus* rates were relatively low (<10%), except in Kuwait, Pakistan and South Korea [30]. Higher fusidic acid resistance rates in CoNS than in *S*. *aureus* has been reported in some European countries (12.5% to 50.0%), the United States (7.2%), Canada (20.0%) and Australia (10.8%) [27, 28]. In Taiwan, the proportion of fusidic acid-resistant *S*. *aureus* at the National Taiwan University Hospital ranged from 3 to 6% [13], which is much lower than the resistance rates in *S*. *epidermidis* (39 to 46%) [15] or in CoNS (48.9%, data from National Taiwan University). However, it has been reported that the novel SCC*fusC* has replaced point-mutated *fusA* as the dominant in fusidic acid resistance mechanism in ST239/SCC*mec*III MRSA in Taiwan after 2008 [13, 17]. Sequence homology analysis suggests that part of SCC*fusC* may originate in *S*. *epidermidis* [17]. In contrast, the major resistance determinant of clinical fusidic acid-resistant *S*. *epidermidis* in Taiwan was *fusB* carried by RIs [15, 16].

The topical use of fusidic acid on skin would select for the *fusB*-family genes in those individuals colonized by *S*. *aureus* and CoNS. Analysis of fusidic acid resistance in nasal carriage *S*. *aureus* isolated from general medical practice patients with non-infectious conditions in Europe revealed that acquired *fusB* and *fusC* were dominant resistant mechanisms [31]. As for CoNS, which are the possible reservoirs for horizontal gene transfer, there have been no reports regarding the fusidic acid resistance determinants or mechanisms in colonizing strains that have not caused diseases in their hosts. Therefore, we examined the fusidic acid resistance genes in colonized staphylococci and compared their genetic environment to those in clinical isolates, and we evaluated the possibility of genetic exchanges between commensal and pathogenic staphylococci.

## Materials and Methods

### Sample collection

A total of 59 healthy, 16- to 18-year-old volunteers with no recent record of hospitalization or diseases from a senior high school in Taipei, Taiwan were enrolled in this study. The isolates were obtained during school hours in August 2010 by drawing the fingers of the right hand (index finger, middle finger and ring finger) with gentle pressure across the surface of mannitol salt agar (Difco Laboratories, Detroit, MI). After incubation at 37°C for 24 h, a total of 853 isolates were collected. This study was approved by the National Taiwan University Hospital Institutional Review Board (201307006RIN), waiving the requirement for written informed consent.

### Identification and genotyping of fusidic acid-resistant staphylococci

The isolates were first screened by subculturing on Mueller-Hinton II agar (Difco Laboratories, Detroit, MI) containing 1 μg/ml fusidic acid. The susceptibility of the growing isolates were tested by the agar dilution method, and the minimal inhibition concentration (MIC) values ≧ 2 μg/ml would be interpreted as fusidic acid-resistant. Species identification was performed by Gram staining, the catalase test and the molecular methods described below. The isolates with no characteristics of staphylococci were excluded from this study. Bacterial DNA was purified with a DNA isolation kit (Puregene, Gentra Systems) according to the manufacturer’s instructions. The following three PCR-based methods were carried out to identify the isolates at the species or subspecies level: (i) *dnaJ* PCR-restriction fragment length polymorphism (RFLP) analysis [32]; (ii) *S*. *epidermidis*-specific PCR [33]; (iii) 16S rRNA gene sequencing. The *dnaJ* PCR-RFLP analysis allows for the differentiation between subspecies pairs of *S*. *capitis*, *S*. *cohnii* and *S*. *hominis* [32].

Pulsed-field gel electrophoresis (PFGE) was performed as previously described [13]. In brief, The DNA was digested with SmaI (New England BioLabs, Ipswich, MA) and then was separated using a CHEF-DRIII apparatus (Bio-Rad Laboratories). PFGE was carried out at 200 V and 12°C for 20 h with the pulse times ranging from 5 to 60 s.

### Antimicrobial susceptibility testing

Antimicrobial susceptibility testing was performed by the agar dilution method according to CLSI 2014 guidelines [34]. Bacterial inocula were prepared by direct colony suspension to a turbidity of 0.5 McFarland standards. A bacterial density of 10^4^ CFU/spot was inoculated onto Mueller-Hinton II agar (BBL) with various concentrations of fusidic acid (0.03 to 256 μg/ml) using a Steers replicator, and the plates were incubated at 35°C for 16 to 20 h. *S*. *aureus* ATCC 29213 was used as the reference strain. The breakpoint used to indicate fusidic acid resistance was ≧ 2 μg/ml [11, 13, 15, 35], although the breakpoint is defined as > 1 μg/ml by EUCAST [36].

### Detection of acquired fusidic acid resistance determinants and their genetic environments

The presence of the acquired fusidic acid resistance determinants *fusB*, *fusC*, *fusD* and *fusF* was detected by PCR as previously described [11, 13]. The *fusB* element on plasmids or located in RIs (integrated into *groEL*, *rpsR* or *smpB*) was determined by PCR using the primers listed in Table 1, and the positions of the primers were indicated in S1 Fig. The presence of SCC*fusC* was detected by PCR using the primer sets listed in Table 1 and the primer sets (B to U) covering almost the entire region as previously described [17]. The schematic diagram of SCC*fusC* mapping was shown in S2A Fig.

**Table 1:** Primers used in this study^a^.

Primer set	Primer name	Sequence (5'-3')	Reference
Detection of RI integrated into *groEL* in *S*. *epidermidis*			
a	S. epi groEL 1213-1232F	CTKGAAGAAGGTATYGTTGC	[15]
	int (I) 109-128F	CGTAAATCAGACGCTAAACA	[15]
b	int (I) 109-128F	CGTAAATCAGACGCTAAACA	[15]
	int (I) 1139-1122R	CTAAACTTGTGGGAAGCG	[15]
c	fusB531-559F	CGGATGGTCAATATGTAAAAAAAGGTGAC	[13]
	185 LA 3R	CTCACAGAGGTTCTATAATGTTGG	[15]
Detection of RI integrated into *smpB* in *S*. *epidermidis*			
d	S.epi ssra407-429 (F)	TCAAGCACTTAAAGAAAAAGCGG	[16]
	int (III) 175–194F	GACGAGTTAGAGGGTATTGG	[16]
e	int (III) 175–194F	GACGAGTTAGAGGGTATTGG	[16]
	int (III) 1087–1068R	TACTAGGGTACAAATGACCG	[16]
f	fusB531-559F	CGGATGGTCAATATGTAAAAAAAGGTGAC	[13]
	S.epi sodium transporter 1146–1168F	TCTCACTATGGATTTAACTTCCG	[16]
Detection of RI integrated into *rpsR* in *S*. *epidermidis*			
g	S epi rpsR 6-24F	AGGTGGACCAAGAAGAGGC	[15]
	int(II) 541-565F	GCTAAACGTAATAACTATTTAGAAG	[15]
h	int(II) 541-565F	GCTAAACGTAATAACTATTTAGAAG	[15]
	int 1061-1042R	GTGTGACGTAATGTGTGCGT	[15]
i	fusB531-559F	CGGATGGTCAATATGTAAAAAAAGGTGAC	[13]
	fusB LA-2R	AATACTCCTGGATGGCGT	[15]
Detection of RI integrated into *groEL* in *S*. *capatis* subsp. *urealyticus* (primer sets a and b also used)			
j	fusB531-559F	CGGATGGTCAATATGTAAAAAAAGGTGAC	[13]
	cap-CWA-R	CYTCMTCTTCGTCAGGAT	This study
Sequencing of ScRI_*fusB*_ (primer sets a, b, and j also used)			
k	7778 PstI up2F	CGCTGATACCTTTGTTGAAC	This study
	fusBR	ACAATGAATGCTATCTCGACA	[13]
j	Sepi 2793up2F	AAAGTGCTGTATGGCGTG	This study
	ri17 284-265R	TCCATAGCATTTAATCCGTG	This study
Detection of the *fusB*-carrying plasmid pUB101 and its relatives			
m	IS257 518-499R	ATATGACGGTGATCTTGCTC	[13]
	fusB 283-254R	AGGTAGTTCAAAAG	[13]
n	IS257 33-52F	GGATGTTATCACTGT	[13]
	fusB 530-558F	CGGATGGTCAATATGTAAAAAAAGGTGAC	[13]
Detection and sequencing SCC*fusC*			
o	orfX-uF	ACTTCGTCTTCGTCATTGG	This study
	hsdR_593R	CTCCAATAAAACATTTGTCCC	[17]
p (inverse PCR)	SAS0044dn382R	GGATTCAGAATGGTTTCC	[17]
	SAS0046_226R	AACCTTCGGTATCATCCG	This study
Sequencing of pseudo SCC and its flanking region			
q (LA PCR)	fusC 162-183F	GGACTTTATTACATCGATTGAC	[13]
	fusC 572-550R	CTGTCATAACAAATGTAATCTCC	[13]
r (inverse PCR)	21429-helicase-R	CGGCTTGAAACTGTAACC	This study
	21429A-copBR	GTATGACAAGTATCGCAGCG	This study
s (inverse PCR)	copB-F	ATACGAGTTGGTGAAACCTTAC	This study
	TFGfusC 7914F	TAACGGTCATTTCACTCG	This study
t	MFS-transF	GAACAGATTTAGCAAAGTCAC	This study
	speG-7F	CTAAGAGCATTAGAGTATAGTG	[17]
u (inverse PCR)	up speG-R	GATTTGTATGAATGGCACTC	This study
	speG 408R	TGTTTTAAATCCTTGTGACTCG	[17]
v	speGR408R	TGTTTTAAATCCTTGTGACTCG	[17]
	hominis-afSCC-R	TTCTTCTGAAACTATCTGCTGG	This study

^a^ The positions of the primers are indicated in S1 Fig (*fusB*-carrying elements) or S2 Fig (*fusC*-carrying elements).

### Sequencing of the *fus* elements ScRI_*fusB*_, SCC*fusC* and pseudo SCC

The sequence of ScRI_*fusB*_ was determined by five PCRs covering the entire region using the primers listed in Table 1 and illustrated in S1D Fig. The sequence of SCC*fusC* was determined by the 21 primer sets used for PCR mapping, and the extreme right region was determined by inverse PCR as shown in S2A Fig. To determine the sequence of pseudo SCC, general PCR, the LA PCR in vitro cloning kit (Takara Shuzo Co.) or inverse PCR were used as shown in Table 1 and S2B Fig. In brief, PCR primers specific for the known sequence were used, and the PCR product was subsequently sequenced. To obtain the full sequence of the corresponding mobile elements, the fragments were used as probes for Southern blot hybridization to determine a suitable restriction enzyme to use for further cloning. The sequence was collected by aligning and combining the amplification fragments obtained by LA PCR or inverse PCR.

### Multilocus sequence typing (MLST)

The MLST was analyzed in 14 *fusB*-positive *S*. *epidermidis* strains and 5 *fusC*-positive *S*. *hominis* subsp. *hominis* strains according to the methods described previously [37, 38]. The new sequence types were deposited in the *S*. *epidermidis* and *S*. *hominis* MLST databases. The eBURST method was used to infer the evolutionary relatedness of STs (http://www.mlst.net).

### Detection of virulence genes associated with invasive infection in *S*. *epidermidis* isolates

We detected the *icaAB* of the *ica* locus, IS256 and *mecA* to discriminate between virulent and non-virulent isolates as previously described [16].

### Nucleotide sequences

The nucleotide sequences of SCC*fusC* of *S*. *hominis* subsp. *hominis* TFGsh1, ScRI_*fusB*_ of *S*. *capitis* subsp. *urealyticus* TFGsc1 and pseudo SCC of *S*. *hominis* subsp. *hominis* TFGsh5-1 have been deposited in the GenBank database under accession numbers AB930126 to AB930128.

## Results

### Species distribution of fusidic acid-resistant staphylococci from hand skin flora

Among the 853 isolates collected from 59 volunteers, a total of 70 isolates recovered from 22 individuals (22/59 = 37.3%) were found to be fusidic acid-resistant staphylococci (MIC values ≧ 2 μg/ml). The isolates obtained from the same person exhibiting identical PFGE patterns were considered to be the same strain. Therefore, 34 fusidic-acid resistant CoNS strains were obtained, and no fusidic acid-resistant *S*. *aureus* was found. Among the 34 fusidic-acid resistant CoNS strains, the most common species was *S*. *epidermidis* (14 strains in 38 isolates), followed by *S*. *cohnii* subsp. *urealyticus* (5 strains in 14 isolates), *S*. *hominis* subsp. *hominis* (5 strains in 8 isolates), *S*. *saprophyticus* (4 strains/isolates), *S*. *capitis* subsp. *urealyticus* (3 strains/isolates), *S*. *warneri* (2 strains/isolates) and *S*. *haemolyticus* (1 strains/isolate) (Table 2). Three *S*. *capitis* subsp. *urealyticus* strains isolated from three different individuals were phylogenetically related because the DNA restriction patterns produced by PFGE had less than two-band differences. Among the 22 individuals who harbored fusidic acid-resistant staphylococci, five were colonized by two species, and one was colonized by four species. There were three individuals who were colonized with multiple strains of *S*. *epidermidis*, and the strains isolated from the same person displayed very limited differences in the PFGE patterns.

**Table 2:** Fusidic acid-resistant CoNS found in 22 individuals.

Individual code	Species	No. of strains (isolates)	Fusidic acid determinant	Location of *fus* element
1	*S*. *epidermidis*	1 (1)	*fusB*	RI integrated into *smpB*
	*S*. *hominis* subsp. *hominis*	1 (1)	*fusC*	SCC*fusC*
2	*S*. *cohnii* subsp. *urealyticus*	1 (1)	*fusF*	ND^a^
	*S*. *saprophyticus*	1 (1)	*fusD*	ND^a^
3	*S*. *epidermidis*	1 (2)	*fusB*	RI integrated into *groEL*
4	*S*. *warneri*	1 (1)	*fusB*	Unknown
5	*S*. *capitis* subsp. *urealyticus*	1 (1)	*fusB*	RI integrated into *groEL*
	*S*. *cohnii* subsp. *urealyticus*	1 (10)	*fusF*	ND^a^
	*S*. *epidermidis*	1 (1)	*fusB*	RI integrated into *groEL*
	*S*. *warneri*	1 (1)	*fusC*	Unknown
6	*S*. *cohnii* subsp. *urealyticus*	1 (1)	*fusF*	ND^a^
	*S*. *epidermidis*	3 (4)	*fusB*	RI integrated into *smpB*
7	*S*. *capitis* subsp. *urealyticus*	1 (1)	*fusB*	RI integrated into *groEL*
	*S*. *epidermidis*	2 (5)	*fusB*	RI integrated into *smpB*
8	*S*. *saprophyticus*	1 (1)	*fusD*	ND^a^
9	*S*. *saprophyticus*	1 (1)	*fusD*	ND^a^
10	*S*. *epidermidis*	1 (1)	*fusB*	Unknown
11	*S*. *cohnii* subsp. *urealyticus*	1 (1)	*fusF*	ND^a^
12	*S*. *epidermidis*	2 (8)	*fusB*	RI integrated into *smpB*
13	*S*. *hominis* subsp. *hominis*	1 (1)	*fusC*	SCC*fusC*
14	*S*. *cohnii* subsp. *urealyticus*	1 (1)	*fusF*	ND^a^
15	*S*. *hominis* subsp. *hominis*	1 (2)	*fusC*	SCC*fusC*
16	*S*. *epidermidis*	1 (1)	*fusB*	Unknown
17	*S*. *capitis* subsp. *urealyticus*	1 (1)	*fusB*	RI integrated into *groEL*
18	*S*. *epidermidis*	1 (14)	*fusB*	RI integrated into *smpB*
19	*S*. *epidermidis*	1 (1)	*fusB*	RI integrated into *smpB*
	*S*. *hominis* subsp. *hominis*	1(1)	*fusC*	Unknown
20	*S*. *hominis* subsp. *hominis*	1 (3)	*fusC*	Pseudo SCC
21	*S*. *haemolyticus*	1 (1)	*fusC*	Unknown
22	*S*. *saprophyticus*	1 (1)	*fusD*	ND^a^

^a^ Not determined because *fusD* and *fusF* have been reported to be intrinsic in the *S*. *saprophyticus* and *S*. *cohnii* subsp. *urealyticus* chromosome, respectively [7, 11].

### Fusidic acid resistance determinants

PCR detection of *fusB*-type genes (*fusB*, *fusC*, *fusD* and *fusF*) was performed on the 34 fusidic acid-resistant staphylococci. As shown in Table 2, all *S*. *epidermidis* and *S*. *capitis* subsp. *urealyticus* isolates possessed *fusB*, whereas the *S*. *hominis* subsp. *hominis* and *S*. *haemolyticus* isolates carried the *fusC* gene. *S*. *warneri* harbored *fusB* or *fusC*. The *fusD* and *fusF* genes were found exclusively in *S*. *saprophyticus* and *S*. *cohnii* subsp. *urealyticus* strains, respectively.

### Genetic environments of *fusB*

The locations of *fusB*, which appears to be associated with mobile genetic elements, were examined by PCR based on the known sequences of RIs or plasmids (Table 1). Of the 14 *S*. *epidermidis* strains, 12 strains carried *fusB* by RIs either integrated into *groEL* (n = 2) or *smpB* (n = 10) (Table 2). The location of *fusB* in the remaining two *S*. *epidermidis* strains and one *S*. *warneri* strain remains unknown.

**Fig 1:**
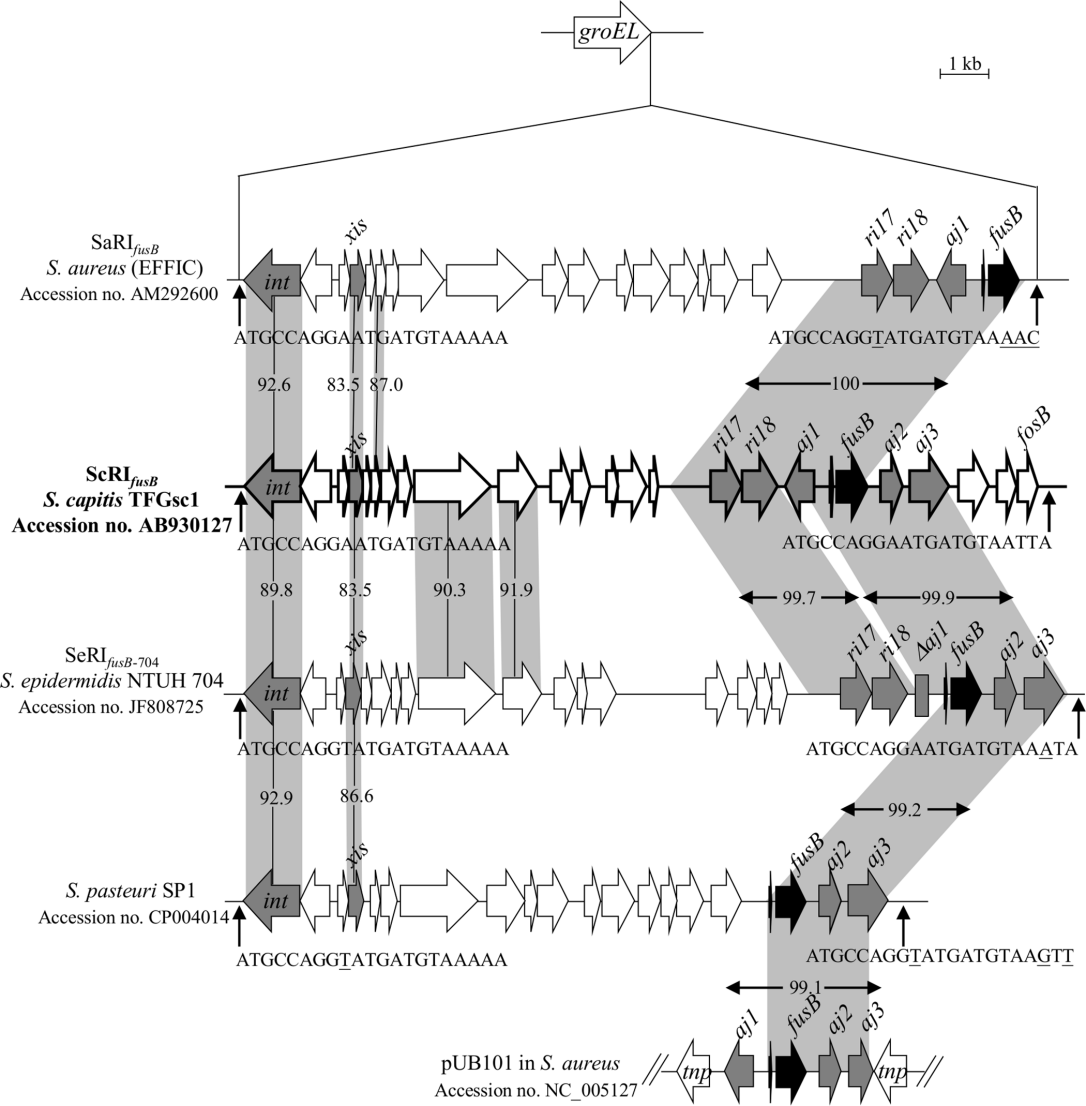
Structure of ScRI_*fusB*_ in *S*. *capitis* subsp. *urealyticus* TFGsc1. ScRI_*fusB*_ was compared to SaRI_*fusB*_, SeRI_fusB-704_, the RI in the *S*. *pasteuri* genome (the above RIs are inserted into *groEL*) and the plasmid pUB101. The ORFs are shown as arrows, and the genes of interest are indicated as grey or black arrows. The homologous regions are shaded, and the numbers in the shadow show the percent homology between the corresponding sequences in comparison to ScRI_*fusB*_. The predicted *att* sites are indicated by vertical arrows. Th divergent nucleotides in the 21-bp *att* sequences are underlined.

The three *S*. *capitis* subsp. *urealyticus* strains were found to acquire *fusB* by a RI integration into *groEL* using PCR primer sets designed in this study (Table 1). Because there is no report on the structure of the *fusB* element in *S*. *capitis*, strain TFGsc1 (isolated from individual No. 5) was subjected to sequencing to confirm the PCR results. Sequence analysis revealed a 16,916-bp RI integrated into *groEL*, which was referred to as ScRI_*fusB*_, where “Sc” signifies “*S*. *capitis*”. ScRI_*fusB*_ had 24 putative open reading frames (ORFs) (Fig 1 and S1 Table). It carried the *ri17*-*ri18*-*aj1*-*fusB*-*aj2*-*aj3* locus, which is always fragmented in other reported mobile genetic elements, such as SaRI_*fusB*_, SeRI_fusB-704_, RI in *S*. *pasteuri* SP1 and pUB101 (Fig 1).

One person (individual No. 5) was colonized by *S*. *capitis* subsp. *urealyticus* and *S*. *epidermidis*. Both of the species carried *fusB*-related RIs with the same integration sites in *groEL*. To know if horizontal gene transfer has occurred between the two species, the structures of the *fusB* element in *S*. *epidermidis* was further analyzed using the resolved sequence of ScRI_*fusB*_ (16,916-bp RI) from the *S*. *capitis* subsp. *urealyticus*. However, PCR mapping revealed that the *fusB* surrounding region in *S*. *epidermidis* was different from ScRI_*fusB*_, indicating the two species did not share the same mobile genetic structures.

### Genotypic analysis of fusidic acid-resistant *S*. *epidermidis*

To further understand the genetic relatedness of the 14 *fusB*-positive *S*. *epidermidis* strains, we determined the sequence types by MLST and the presence of genes associated with invasive infections (*icaAB*, IS*256* and *mecA*) [16]. As shown in Table 3, 7 sequence types were identified and were clustered by eBURST algorithm into three clonal complexes (CCs): CC2 (n = 7), CC365 (n = 4) and a CC with no predicted founder (n = 3). The prevalence of *icaAB*, IS*256* and *mecA* was low for the 14 *S*. *epidermidis* strains. The strains isolated from individual No. 6, 10 and 16 were ST57, but exhibited differences in the virulence gene patterns and the mechanism by which the *fusB* elements were acquired. The strains isolated from individuals No. 1 and 5 were ST438 and shared identical virulence gene patterns, although they acquired *fusB*-carrying RIs integrated into different sites. The strain isolated from individual No. 18 was ST-NT2, which is a single locus variant of ST-NT3 found in a strain isolated from individual No. 19. The *S*. *epidermidis* strains isolated from the same individual (No. 6, 7 and 12) shared identical genetic patterns within the same person.

**Table 3:** Genetic characteristics of *S*. *epidermidis* carrying *fusB* elements at different integration sites.

RI integration site	Individual code	Clonal complex	MLST profile	Genes associated with invasive infections		
				*icaAB*	IS*256*	*mecA*
*smpB*	1	365	438 (3-25-5-5-11-4-11)	-	-	-
	6 ^a^	2	57 (1-1-1-1-2-1-1)	-	-	+
	7 ^a^	2	194 (7-1-2-2-4-1-13)	+	-	-
	12 ^a^	NPF^c^	NT1^b^ (3-16-9-5-3-x1^b^-5)	-	-	-
	18	365	NT2^b^ (3-25-5-5-11-x2^b^-20)	-	-	-
	19	365	NT3^b^ (3-25-5-5-11-4-20)	-	-	-
*groEL*	3	NPF^c^	208 (3-3-13-5-7-4-4)	-	-	-
	5	365	438 (3-25-5-5-11-4-11)	-	-	-
Unknown	10	2	57 (1-1-1-1-2-1-1)	-	-	-
	16	2	57 (1-1-1-1-2-1-1)	-	+	+

^a^ The multiple strains obtained from the same individual (No. 6, No. 7 or No. 12) display identical patterns in each person.

^b^ Novel allele or ST found in this study. NT3 represents novel combination of known alleles, while NT1 and NT2 represent combinations containing novel allele sequences of *tpi*. The novel allele sequences and ST have been submitted to the *S*. *epidermidis* MLST database (http://sepidermidis.mlst.net). The two novel allele sequences of *tpi* can be found in S1 Text.

^c^ NPF: the ST-NT1 and ST208 were clustered in a clonal complex with no predicted founder.

### Genetic environments of *fusC*

**Fig 2:**
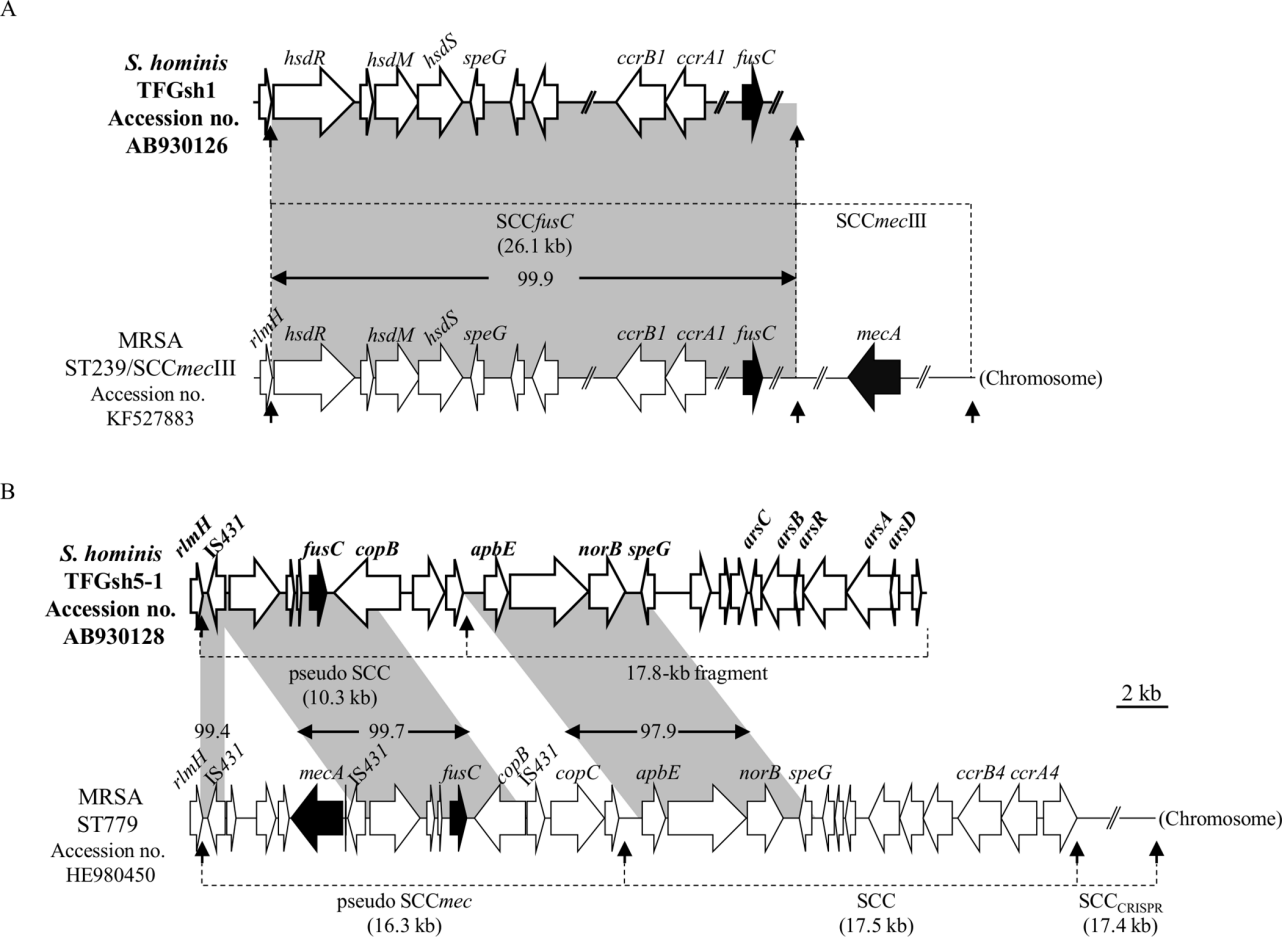
Genetic organization of *fusC-*related elements in *S*. *hominis* subsp. *hominis*. (A) Schematic maps of SCC*fusC* in strain TFGsh1 and (B) the composite SCC structure in strain TFGsh5-1 are shown. The ORFs are shown as arrows, and the drug resistance genes *fusC* and *mecA* are shown as black arrows. The homologous regions are shaded, and the numbers in the shadow show the percent homology. The *att* sequence is indicated by vertical arrows.

A total of 7 *fusC*-positive strains, including 5 *S*. *hominis* subsp. *hominis*, 1 *S*. *haemolyticus*, and 1 *S*. *warneri*, were found (Table 2). As *fusC* was mostly found within the SCC structure in staphylococci [17–20], we first detected the *ccr* genes (encoding cassette chromosome recombinases) by PCR. Three *S*. *hominis* subsp. *hominis* strains and 1 *S*. *warneri* strain were positive for *ccrA1B1*. Further PCR mapping revealed that all three *ccrA1B1*-positive *S*. *hominis* strains carried SCC*fusC*. Because the SCC*fusC* has only been reported in ST239/SCC*mec*III MRSA [17], we subsequently determined the sequence of SCC*fusC* in the *S*. *hominis* subsp. *hominis* strain TFGsh1 to confirm the PCR mapping results. Nucleotide sequence analysis indicated 99.9% similarity (16 bp mismatch) compared to SCC*fusC* in ST239/SCC*mec*III MRSA strain NTUH-4729 (Fig 2A and S2 Table).

To determine the location and structure of the *fusC* element in the other two *S*. *hominis* subsp. *hominis* strains which do not carry known *ccrAB* or *ccrC*, the flanking region of *fusC* in one strain (TFGsh5-1) was cloned and sequencing. The result revealed that *fusC* in TFGsh5-1was present in a pseudo SCC structure and was inserted at the 3′-end of *rlmH* (Fig 2B and S3 Table). The pseudo SCC is 10,252-bp in length and consists of 8 ORFs. The IS*431*-*dinA*-*orf*-*orf*-*fusC*-*copB* locus (partial *copB* because 1 to 575 bp was truncated in ST779 MRSA) showed 99.7% nucleotide sequence similarity to the pseudo SCC*mec* in ST779 MRSA. A 17.8-kb fragment was found immediately downstream of the pseudo SCC. The region from the second *att* site to *speG* showed 97.9% nucleotide sequence similarity to the SCC in ST779 MRSA.

For another *fusC*-positive, *ccr*-negative *S*. *hominis* subsp. *hominis* strain in individual No. 19, the *dinA*-*orf*-*orf*-*fusC*-*copB* locus was detected, but the chromosome/plasmid location remains unknown because no amplification product could be generated between *rlmH* and the *fusC* flanking region.

For *fusC*-positive *S*. *warneri* (n = 1) and *S*. *haemolyticus* (n = 1), the genetic environments of *fusC* were unknown.

### Genotyping of fusidic acid-resistant *S*. *hominis* subsp. *hominis*

MLST was carried out to understand the phylogenetic relationships between the five fusidic acid-resistant *S*. *hominis* subsp. *hominis* strains. As shown in Table 4, two SCC*fusC*-carrying strains isolated from individuals No. 13 and 15 shared the same sequence type. The other strains were of diverse genetic backgrounds.

**Table 4:** Genotypes of *fusC*-positive *S*. *hominis* subsp. *hominis*.

Structure of *fusC* element	Individual code	MLST profile
SCC*fusC*	1	41^a^ (11^a^-2-13-1-6-3)
	13	42^a^ (2^a^-5-13-4-6-3)
	15	42^a^ (2^a^-5-13-4-6-3)
Pseudo SCC	20	44^a^ (17^a^-5-13-4-7-3)
Unknown	19	43^a^ (1^a^-3-3-12^a^-6-6^a^)

^a^ Novel allele or ST found in this study.

## Discussion

This is the first report to study fusidic acid resistance in staphylococci among the skin flora of healthy volunteers. In this study, we did not find fusidic acid-resistant *S*. *aureus* among the skin flora, although we have isolated fusidic acid-susceptible *S*. *aureus* (data not shown). The result is in accord with the low resistance rate to fusidic acid in clinical isolates of *S*. *aureus* in Taiwan (0 to 6%) [15, 30]. The overall resistance rate of fusidic acid in CoNS from skin flora in the community was 37.3%, which was lower than those isolated from hospitalized patients in Taiwan (48.9%, introduction) but is still higher than CoNS isolated from hospitals in the United States (7.2%), Canada (20.0%), Australia (10.8%) or some European countries (Greece, Israel, Italy, Poland, Spain and Turkey, 12.5% to 32.0%) [27, 28].

In the present study, the breakpoint of 2 μg/ml was used to interpret fusidic acid resistance as we used before [11,13,15], instead of ≥1 μg/ml recommended by the EUCAST [36]. Therefore, the overall fusidic acid resistance rate would probably slightly increase if the EUCAST breakpoint is applied.

The fusidic acid resistance among 34 resistant strains was mostly mediated by *fus*B and *fusC*. The *fusB* genes in the present study were mainly chromosomally encoded within resistance islands (RIs) in specific chromosomal locations, while *fusC* was carried by different SCC elements, including the previously described SCC*fusC* and a new pseudo SCC element. The *fusB*-related RI elements have been reported in *S*. *aureus*, *S*. *pasteuri*, and *S*. *epidermidis* clinical isolates [15,16]. In the present study, we first found that *S*. *capitis* subsp. *urealyticus* carried *fusB*-RI. Both RIs and pathogenicity islands (PIs) are phage-related chromosomal islands that produce phage-like infectious particles by hijacking the capsids of phages [39]. To date, several PI-related accessory virulence genes have been described, such as *tst* (encoding toxic shock syndrome toxin) and *seb* (encoding staphylococcal enterotoxin B). Orthologues of these genes have been reported in different PIs, but most of them are still restricted in the *S*. *aureus* genome [39]. Unlike the PI-related virulence genes, the *fusB* was found not only in the RIs of four different *Staphylococcus* species but also on a *S*. *aureus*-derived plasmid, pUB101 [12, 14–16] (Fig 1). Comparison of the immediate flanking regions of *fusB* among the different species revealed high sequence similarities (> 99.1%), even though some deletions were observed (Fig 1). The low G + C content (26.6%) of the ScRI_*fusB*_ *ri17*-*ri18*-*aj1*-*fusB*-*aj2*-*aj3* locus compared to the *S*. *capitis* genome (32.76%) [40] implies that the element may originate in other bacterial species and then disseminate into staphylococci by RIs or plasmids.

We have previously reported a novel emerging SCC*fusC* in fusidic acid-resistant ST239/SCC*mec*III MRSA in Taiwan [17]. In the present study, we unexpectedly found three of five *S*. *hominis* strains carried the SCC*fusC*, and the sequence of SCC*fusC* in strain TFGsh1 was nearly identical to that in ST239/SCC*mec*III MRSA. To date only *S*. *hominis* and no other CoNS are known to carry SCC*fusC*. Thus, the commensal *S*. *hominis* may act as an important reservoir for horizontal gene transfer for the dissemination of *fusC* to ST239/SCC*mec*III MRSA in Taiwan, although the origin of SCC*fusC* remains to be explored.

The other *fusC*-related structure found in the present study was a pseudo SCC element. The *fusC* flanking region in the pseudo SCC of *S*. *hominis* displayed a high sequence similarity to flanking region of the pseudo SCC*mec* in ST779 MRSA (Fig 2B) except the lack of a 4.7-kb fragment harboring *mecA*, which was flanked by IS*431* direct repeats. Thus, the *S*. *hominis* pseudo SCC may result from replacement of the *mecA* element by *fusC* via homologous recombination between the two IS*431* elements, which belongs to the IS*6* family (e.g., IS*431* and IS*1216V*) [41]. Another IS*431* located in right side of the MRSA ST779 pseudo SCC*mec*, which gave rise to 5′-end truncation of *copB*, may also lead to differences between pseudo SCC and pseudo SCC*mec*. In *S*. *hominis* strain TFGsh5-1, a 17.8-kb fragment was demarcated from the pseudo SCC by an *att* sequence (Fig 2B). The 17.8-kb fragment carried virulence-related genes, *speG* and *arsCBRAD*, which are usually components of mobile genetic elements but are not present in the core chromosome of *S*. *hominis* [26, 42]. Hence, this 17.8-kb fragment behaved just like an SCC, although it did not contain the *ccr* genes. The mosaic structures of pseudo SCC implied that multiple recombination events have occurred after acquisition of the foreign genetic element into *S*. *hominis* or *S*. *aureus*.

Multiple *S*. *epidermidis* strains isolated from the same person exhibited identical sequence types as well as virulence gene patterns associated with invasive infections (Table 3); only limited differences were observed in the PFGE profiles. They also acquired *fusB* elements that were integrated into the same site (Table 2). This suggested that these strains may have acquired *fusB*-carrying RIs once and then undergone evolutionary changes to produce slightly different PFGE patterns in the same host.

Previous studies have shown that the CC2 was the most common *S*. *epidermidis* in both hospital (87.3% to 100%) and community (58%) [43–46]. However, prevalence of *icaAB*, IS*256* and *mecA* in CC2 *S*. *epidermidis* isolated from community environment was lower than that in clinical isolates even they shared similar genetic background [44,46]. For the 14 *fusB*-positive *S*. *epidermidis* strains which were CC2 or other minor clonal lineages, the overall prevalence of *icaAB*, IS*256* and *mecA* was low (Table 3). It implies that the *fusB*-positive *S*. *epidermidis* in skin flora is not originated in hospital.

In conclusion, fusidic acid resistance in commensal staphylococci was found to be mainly mediated by the *fusB*-family genes. At least four types of mobile genetic elements carrying *fusB* or *fusC* were responsible for the fusidic acid resistance in CoNS, suggesting multiple events of horizontal gene transfer have occurred among various species or lineages in community. The structures of the acquired resistance elements were similar to the structures in clinical isolates, implying that commensals may act as reservoir for the pathogens. Furthermore, the high similarities of SCC*fusC* provide evidence for possible horizontal transfer between commensal *S*. *hominis* and ST239/SCC*mec*III MRSA.

## Supporting Information

S1 Fig Carton representation of PCR mapping and sequencing for *fusB*-carrying elements. Schematic maps of RI in *S*. *epidermidis* integrated into *groEL* (A), *smpB* (B) and *rpsR* (C), RI in *S*. *capitis* subsp. *urealyticus* integrated into (D) *groEL* and plasmid pUB101 (E) are shown. The arrows below the structures indicate PCR primers, which are listed in Table 1.
(PDF)

S2 Fig Carton representation of PCR mapping and sequencing for fusC-carrying elements. Schematic maps for SCC*fusC* (A) and pseudo SCC with its flanking region (B) are shown. The arrows below the structures indicate PCR primers, which are listed in Table 1.
(PDF)

S1 Table Genetic organization of ScRI_*fusB*_. (XLS)

S2 Table Genetic organization of SCC*fusC*. (XLS)

S3 Table Genetic organization of pseudo SCC and its flanking region. (XLS)

S1 Text Novel *tpi* allele sequences of *S*. *epidermidis* found in this study. (TXT)
