# Supplementary material for: Skin Commensal Staphylococci May Act as Reservoir for Fusidic Acid Resistance Genes
Source: PLoS One. 2015 Nov 18;10(11):e0143106. doi: 10.1371/journal.pone.0143106 (PMC4651549; doi:10.1371/journal.pone.0143106)

S1 FIG.

A (RI integrated into *groEL* in *S. epidermidis*)

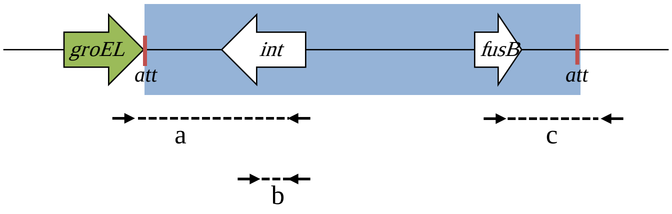

B (RI integrated into *smpB* in *S. epidermidis*)

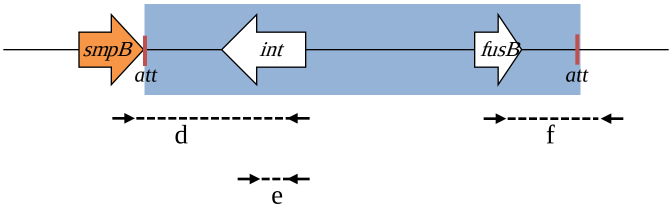

C (RI integrated into *rpsR* in *S. epidermidis*)

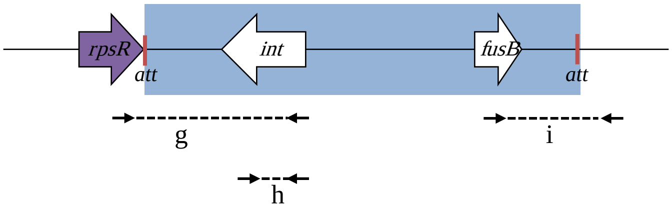

D (RI integrated into *groEL* in *S. capitis* subsp. *urealyticus)*

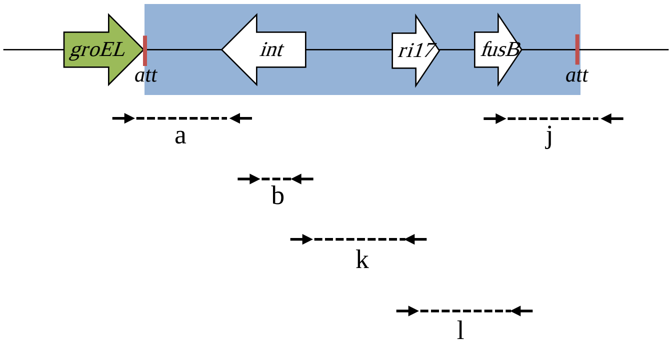

E (Plasmid)

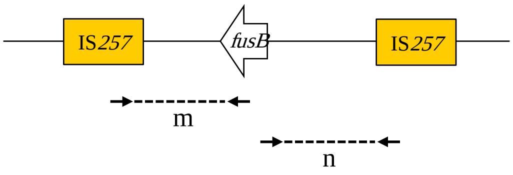

Supplement: S1 Fig — Schematic maps of RI in S. epidermidis integrated into groEL (A), smpB (B) and rpsR (C), RI in S. capitis subsp. urealyticus integrated into (D) groEL and plasmid pUB101 (E) are shown. The arrows below the structures indicate PCR primers, which are listed in Table 1. (PDF) [file pone.0143106.s001.pdf]
