# Supplementary figures and images for: Skin Commensal Staphylococci May Act as Reservoir for Fusidic Acid Resistance Genes
Source: PLoS One. 2015 Nov 18;10(11):e0143106. doi: 10.1371/journal.pone.0143106 (PMC4651549; doi:10.1371/journal.pone.0143106)

S2 FIG.

A (SCC*fusC*)

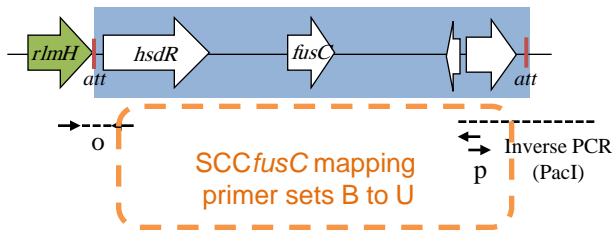

B (Pseudo SCC and the 17.8-kb fragment)

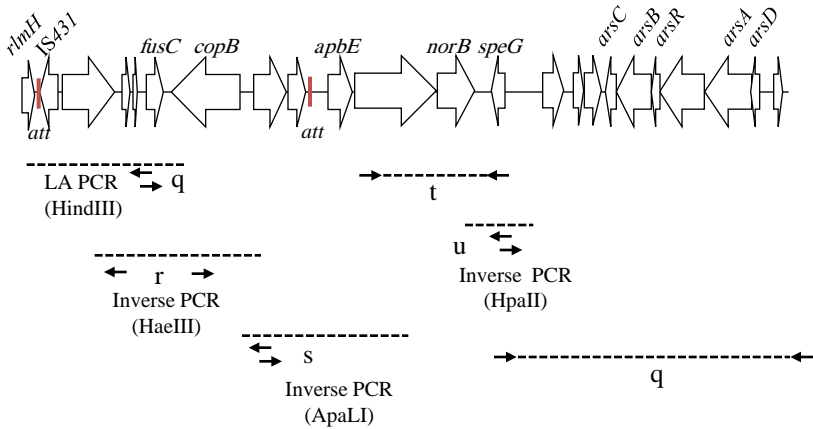

Supplement: S2 Fig — Schematic maps for SCCfusC (A) and pseudo SCC with its flanking region (B) are shown. The arrows below the structures indicate PCR primers, which are listed in Table 1. (PDF) [file pone.0143106.s002.pdf]
